# Measuring diffusion exchange across the cell membrane with DEXSY (Diffusion Exchange Spectroscopy)

**DOI:** 10.1002/mrm.28207

**Published:** 2020-02-14

**Authors:** James O. Breen‐Norris, Bernard Siow, Claire Walsh, Ben Hipwell, Ioana Hill, Thomas Roberts, Matt G. Hall, Mark F. Lythgoe, Andrada Ianus, Daniel C. Alexander, Simon Walker‐Samuel

**Affiliations:** ^1^ Centre for Advanced Biomedical Imaging Division of Medicine University College London London UK; ^2^ Microstructure Imaging Group Centre for Medical Imaging Computing University College London London UK; ^3^ The Francis Crick Institute London UK; ^4^ UCL Great Ormond Street Institute for Child Health University College London London UK

**Keywords:** cell membrane permeability, DEXSY, Diffusion exchange, FEXSY

## Abstract

**Introduction:**

To combine numerical simulations, in vitro and in vivo experiments to evaluate the feasibility of measuring diffusion exchange across the cell membrane with diffusion exchange spectroscopy (DEXSY).

**Methods:**

DEXSY acquisitions were simulated over a range of permeabilities in nerve tissue and yeast substrates. In vitro measurements were performed in a yeast substrate and in vivo measurements in mouse tumor xenograft models, all at 9.4 T.

**Results:**

Diffusion exchange was observed in simulations over a physiologically relevant range of cell permeability values. In vitro and in vivo measures also provided evidence of diffusion exchange, which was quantified with the Diffusion Exchange Index (DEI).

**Conclusions:**

Our findings provide preliminary evidence that DEXSY can be used to make in vivo measurements of diffusion exchange and cell membrane permeability.

## 1 INTRODUCTION

Water is exchanged between intracellular and extracellular compartments either via direct diffusion across the lipid bilayer^1^ or via integral aquaporin membrane proteins.^1^ Both can be modified in diseases such as cancer,^2^, ^3^ and disruption of the cell membrane causes permeability changes during apoptosis, oncosis, and necrosis.^4^, ^5^, ^6^, ^7^ Thus, measurements of water exchange could provide useful biomarkers of disease progression and response to treatment in cancer, alongside other pathologies such as those responsible for neurodegeneration.^8^

Diffusion MRI is widely used in oncology to measure tissue microstructural properties such as cell density, detecting tumors and characterizing response to therapy. Likewise, diffusion‐weighted imaging has been used to map aquaporin reporter gene expression,^9^ and machine learning approaches for measuring cell membrane permeability have also been trialled.^10^ Other, more specialized MRI techniques have been developed to enable changes in water exchange to be measured, including FEXSY (filter exchange spectroscopy)^11^ and diffusion exchange spectroscopy (DEXSY).^12^ FEXI (filter exchange imaging), the imaging version of FEXSY, provides estimates of exchange using a two‐compartment exchange model.^13^ The techniques both use double diffusion‐encoding schemes to identify water exchange between biological compartments with different diffusion properties.^12^ FEXI estimates of exchange have been linked to gene expression, such as the relationship between the apparent exchange rate and UT‐B transporter expression.^14^ However, FEXI estimates of exchange assume a mono‐exponential exchange between two compartments, which is computationally tractable but may under represent the complexity of biological tissue. For this to be evaluated, techniques such as DEXSY could be used to fully investigate this relationship. Unlike FEXI, DEXSY has not yet been trialed in vivo, and could provide more extensive coverage of diffusion parameter space and a model‐free method for estimating water exchange.

In this study, we have used numerical simulations to assess the feasibility of using DEXSY to measure diffusion exchange across the cell membrane in nerve bundles and yeast, and compared the results with corresponding simulations using FEXSY and diffusion ordered spectroscopy (DOSY). Our rationale for this approach was that these simple models of nerve bundles are a well‐established substrate for diffusion simulations, while the results of yeast simulations can be validated in vitro. We also validate our yeast simulations with in vitro measurements of diffusion exchange across the cell membrane using DEXSY.^12^, ^15^ Here, yeast was chosen as it is a well‐established, generalizable model for cellular biology studies in eukaryotes.^16^ Finally, we acquired DEXSY data from tumor xenograft models to demonstrate the feasibility of measuring diffusion‐diffusion exchange in vivo. Through this series of experiments, we were therefore able to link together and co‐validate in silico, in vitro, and in vivo data.

## 2 METHODS

**Figure 1:**
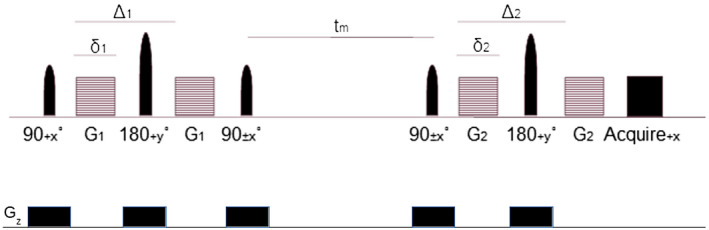
The pulse sequence used in DEXSY, $G_{z}$ Slice encoding gradient, diffusion‐encoding gradient strength, $G_{1}$, $G_{2}$, the mixing time, $t_{m}$, the duration of the diffusion encoding gradients $\delta_{1}$, $\delta_{2}$, and the diffusion times $\Delta_{1}$, $\Delta_{2}$

The DEXSY pulse sequence used to measure diffusion exchange in this study incorporates two pairs of diffusion‐encoding gradients, $G_{1}$ and $G_{2}$ (Figure 1). These are separated by a mixing time, $t_{m}$, which allows diffusion exchange to occur. An inverse Laplace transform is applied to the DEXSY signal to produce a distribution of diffusion coefficients. This can be displayed as a 2D spectrum in which diffusivities encoded with $G_{1}$ are plotted against diffusivities encoded with $G_{2}$. Peaks that lie along the diagonal of these diffusion‐diffusion exchange spectra represent spins exhibiting the same diffusivity during both sets of diffusion encoding gradients, whereas off‐diagonal peaks represent spins that have exchanged between two different diffusion environments.^12^, ^17^ The signal equation for the DEXSY sequence is^17^:

(1) $$\frac{S}{S_{0}}\;=\;\sum p\left(D_{1},\;D_{2}\right)e^{\left(-b_{1}D_{1}\right)}e^{\left(-b_{2}D_{2}\right)}$$

where subscripts correspond to parameters associated with either $G_{1}$ or $G_{2}$. The summation is across all spins in the system. For a pair of gradient pulses, $b\;=\;\gamma^{2}\delta^{2}G^{2}\left(\Delta -\delta /3\right)$, where *γ* is the gyromagnetic ratio, G is the gradient strength, *δ* is the duration of the diffusion encoding gradient, Δ is the diffusion time; D is the distribution of diffusivities, *S* is the acquired signal $S_{0}$ is the signal acquired with no diffusion encoding gradients; *p* is the probability of the contribution to the signals from $D_{1}$ and $D_{2}$. The DEI (Diffusion Exchange Index) is defined here as the ratio of the diffusion exchange signal (sum of the volume of the exchange peaks) to the nonexchange signal (sum of the volume of the intracellular and extracellular peaks), and is proposed as a normalized measure of diffusion exchange.

In FEXI, a similar pulse sequence is used. However, the first gradient pair has a fixed magnitude and is used as a filter, while the mixing time is varied. An estimate of exchange is then calculated from the data acquired using a mono‐exponential two compartment exchange model.^13^ In DOSY acquisitions, chemical shift spectra are acquired in combination with a range of diffusion encoding gradients, allowing the diffusivities of specific molecules to be measured.^18^

### 2.1 Simulations

We simulated measurements of diffusion exchange across the cell membrane using DEXSY and FEXSY in two substrates: (a) a substrate modeling nerve bundles and (b) a yeast substrate. Yeast was used as it is a well‐established model for eukaryotic cells^16^ and enabled us to validate our simulations with in vitro measurements in yeast.

We modified the corpus callosum nerve bundle model used by Alexander et al,^19^, ^20^ to include permeable cell membranes. The nerve bundle model uses a gamma distribution of cylinder radii with a shape parameter of 5.3316, a scale parameter of $1.0242\;\times \;10^{-7}$ m with 100 cylinders and a lattice size of $1.65\;\times \;10^{-5}$ m.

In this study, the probability of walkers crossing the cell membrane was adjusted to include a wide range of permeabilities, which exceeded the range previously measured in a variety of cells.^21^ For this, the probability of a random walker crossing a cell membrane was varied between 0 and 0.1. The 25 probabilities were centered at 0.003, which corresponds to a physiological cell membrane permeability of 1.0 μm/s, and is equivalent to that measured in a rat brain axon.^21^ There were 10 probabilities evenly spaced between 0.0001 and 0.00055. To mimic a yeast suspension, a substrate consisting of 500 spheres (5 μm diameter) with a packing fraction of 0.62 was used.^22^ The probability of walkers crossing the cell membrane is directly proportional to the permeability for a given substrate. The relationship is given by the following equation:

(2) $$p\;=\;k\sqrt{\frac{6dt}{D}}$$

where *p* is the probability of a walker crossing the cell membrane, *k* is the permeability *dt* is the time step and *D* is the diffusivity.^23^

Monte Carlo simulations were performed using CAMINO^24^ with 100 000 walkers, a duration of 400 ms, diffusivity of $2.0\;\times \;10^{-9}\;m^{2}/s$ and 16 000 time steps, in both yeast and nerve tissue substrates. DEXSY and FEXSY signals were simulated in order to determine their relationship with cell membrane permeability.

Parameters for simulated DEXSY acquisitions included *δ* = 15 ms, Δ = 17 ms and $t_{m}\;=\;100$ ms, $G_{1}$ and $G_{2}\;=\;0\text{-}900$ mT/m in 16 × 16 linearly spaced steps. A DOSY^18^ acquisition was also simulated with the following parameters: *δ* = 15 ms, Δ = 17 ms and encoding gradients set to vary between 0 and 900 mT/m in 256 steps. The DOSY is used to validate the accuracy of the DEXSY diffusion measurements. Simulated FEXSY acquisitions included the following parameters: *δ* = 15 ms, Δ = 17 ms and a filter gradient strength of 68 mT/m and an encoding strength varying between 0−68 mT/m in 9 steps with $t_{m}\;=\;0,\;10,\;100,\;200,\;300$ ms.

Two‐dimensional inverse Laplace software^25^ was used to process the DEXSY and DOSY raw data, to produce diffusion‐diffusion exchange plots to summarize DEXSY data, and diffusion spectra for DOSY. The AXR (apparent exchange rate) parameter was estimated from FEXSY simulations, following the method of Nilsson et al.^26^

### 2.2 In vitro experiments

In vitro data were acquired to validate our in silico DEXSY measurements of diffusion‐diffusion exchange in a yeast substrate. The data were acquired using a 20 cm horizontal bore 9.4 T Varian scanner with a 26 mm Rapid RF coil and 1000 mT/m gradient inserts, with a slice selective DEXSY sequence. Two samples were used, consisting of 15 mL falcon tubes containing a suspension of l’hirondelle cake yeast in PBS (18 *g* and 22 *g* in 10 mL PBS, respectively). The two samples were scanned using different DEXSY scan parameters sensitive to different diffusion lengths (10 μm and 9 μm). Data were acquired from sample 1 with DEXSY scan parameters included *δ* = 15 ms, Δ = 17 ms, $t_{m}\;=\;200$ ms, $G_{1}$ and $G_{2}$  = 0‐640 mT/m in 16 × 16 linearly spaced steps. For sample 2, with DEXSY scan parameters included *δ* = 9 ms, Δ = 14 ms, $t_{m}\;=\;200$ ms, $G_{1}$ and $G_{2}\;=\;0\text{-}640$ mT/m in 16 × 16 linearly spaced steps. In each case, the slice used to acquire the data included the whole falcon tube.

### 2.3 In vivo experiments

All in vivo experiments were performed in accordance with the UK Home Office Animals Scientific Procedures Act, 1986 and United Kingdom Coordinating Committee on Cancer Research (UKCCCR) guidelines.^27^ CD‐1 mice were inoculated with 3 million SW1222 cells in their left flank, in order to create a subcutaneous xenograft tumor model. Data were acquired using a 20 cm horizontal bore 9.4 T Varian scanner with a 39 mm Rapid RF coil and 400 mT/m gradient inserts, with a slice selective DEXSY sequence.

A subcutaneous xenograft model was chosen for two reasons: first, our primary interest is in measuring cell membrane permeability in cancer; and secondly because subcutaneous tumor xenografts can easily be captured in a single slice without contamination from other tissues. This approach is less straightforward in normal mouse organs which, with a slice‐selective DEXSY sequence, would exhibit significant partial volume artifacts, making its interpretation challenging. This is even the case in the brain where the skull, in which multiple tissues (eg, ventricles and air cavities) would be included in the slice. Each slice was positioned coronally through the tumor and each slice thickness corresponded to the depth of the tumor being scanned. DEXSY scan parameters included *δ* = 15 ms, Δ = 17 ms, $t_{m}\;=\;200$ ms, $G_{1}$ and $G_{2}\;=$ 0‐640 mT/m in 16 × 16 linearly spaced steps. Mice were scanned under anesthetic with a mixture consisting of 1%‐2.5% isoflurane in 1 L/min of oxygen. The four scans presented here are acquired from slices that included the whole tumor and avoided the surrounding tissue. The cohort initially contained 5 mice, all of which were scanned at the first time point. Due to time constraints and excessive tumor growth, three of those mice were repeat scanned at the second time point.

## 3 RESULTS

### 3.1 Simulations

**Figure 2:**
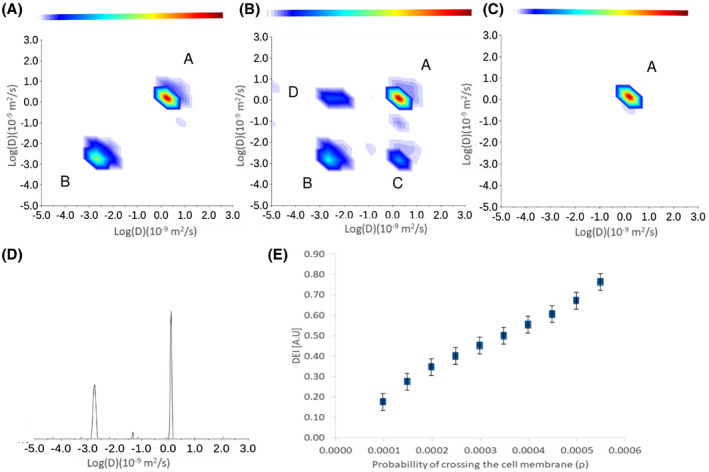
Results of diffusion exchange simulations carried out in a nerve tissue substrate. DEXSY diffusion‐diffusion exchange plots are shown for *p*  = 0.0 (no exchange) (A), *P* = .0003 (B) and *P* = .001 (C). Labeled peaks correspond to extracellular diffusion (A), intracellular diffusion (B), extracellular to intracellular exchange (C), and intracellular to extracellular exchange (D). D, Results of DOSY simulations in the nerve tissue substrate, with *P* = .0 (no exchange). E, DEI vs permeability for a range of permeability values with standard error bars

The results of our simulations of diffusion exchange in a substrate mimicking nerve bundles are shown in Figure 2. In DEXSY diffusion‐diffusion plots, diagonal peaks represent diffusion within a single discrete compartment (here, intracellular and extracellular) and cross‐peaks represent diffusion exchange between compartments. Figure 2A shows a diffusion‐diffusion exchange plot from a simulation carried out in a nervous tissue substrate with *P* = .0 (no exchange). Two peaks can been seen, labeled A and B, positioned on the line of identity; B is associated with intracellular diffusion, due to its lower diffusivity caused by restricted diffusion, and A is associated with extracellular diffusion, due to its higher diffusivity corresponding to hindered diffusion. The diffusivity of peak A is lower than the inherent diffusivity of the simulation ( $2.0\;\times \;10^{-9}\;m^{2}/s$) due to the hindered diffusion environment.

Figure 2B shows a diffusion‐diffusion exchange plot from a simulation carried out in the same substrate at *P* = .0003. Two additional peaks can be observed, labeled C and D, associated with exchange between intracellular and extracellular compartments. Peak B is split and there is an additional spurious peak in the bottom left hand corner. These are assumed to be artifacts introduced by the inverse Laplace transform algorithm.^28^ Figure 2C shows a diffusion‐diffusion exchange plot from a simulation carried out in the same substrate, with *P* = .001. Due to the high permeability, a single peak can be observed, corresponding to the averaged contributions of intracellular and extracellular diffusion. Figure 2D shows a DOSY plot from the same simulation as shown in Figure 2A. This spectrum shows that diffusivities measured with DOSY reflect a projection along the line of identity in the DEXSY diffusion‐diffusion plot, as would be expected. The lower diffusion peak is broader in the off diagonal direction and narrower in the diagonal direction suggesting that the diffusion is restricted.

The DEI is our proposed measure of diffusion exchange rate from DEXSY. Figure 2E shows DEI plotted against permeability in the range corresponding to *P* = .0001 to .00055 (a permeability of 0.37 to 2.0 μm/s), which shows a monotonic increase, and a Spearman’s rank correlation coefficient of 1 (*P* < .05).

**Figure 3:**
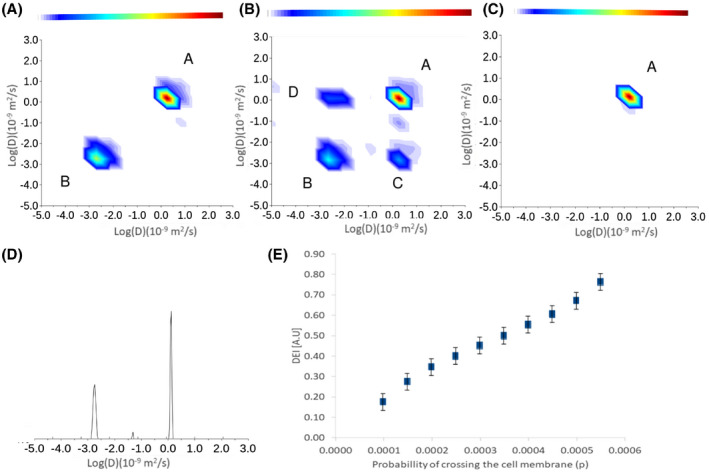
Results of diffusion exchange simulations carried out in the yeast substrate. DEXSY diffusion‐diffusion exchange plots are shown for exchange probabilities of *P* = .0 (A), *P* = .0003 (B), and *P* = .1 (C). As in previous figures, labeled peaks correspond to extracellular diffusion (A), intracellular diffusion (B), extracellular to intracellular exchange (C), and intracellular to extracellular exchange (D). D, Results of DOSY simulations in the yeast substrate, with *P* = .0 (no exchange). E, DEI plotted against permeability

The results of our simulations of diffusion exchange in a yeast substrate are shown in Figure 3. Figure 3A shows a diffusion‐diffusion exchange plot from a simulation carried out in the yeast substrate with *P* = .0 (no exchange). As in the nerve substrate, two peaks can be seen on the line of identity, correpsonding to extracellular and intracellular diffusion (A and B, with low and high diffusivity, respectively). The diffusivity of peak A is lower than the inherent diffusivity of the simulation ( $2.0\;\times \;10^{-9}\;m^{2}/s$) due to the hindered diffusion environment.

Figure 3B shows a diffusion‐diffusion exchange plot from a simulation carried out in the same yeast substrate with *P* = .0003. Here, as in the nerve substrate, an additional two peaks can be observed which correspond to diffusion exchange (C and D). Figure 3C shows a diffusion‐diffusion exchange plot from a simulation carried out in the same substrate at *P* = .1. Due to the high permeability the peaks all merge into a single diffusion peak, coincident with the location of the extracellular peak.

Figure 3D shows a DOSY plot from the same simulation as shown in Figure 3A it shows diffusion measured with DOSY is equivalent to the diffusion along the line of identity as measured with DEXSY. Figure 3E shows DEI plotted against permeability in the range in which four peaks can be found which corresponds to a range of *P* = .0001 to .00055, the relationship is clearly monotonic, with a Spearman’s rank correlation coefficient of 1 (*P* < .05).

**Figure 4:**
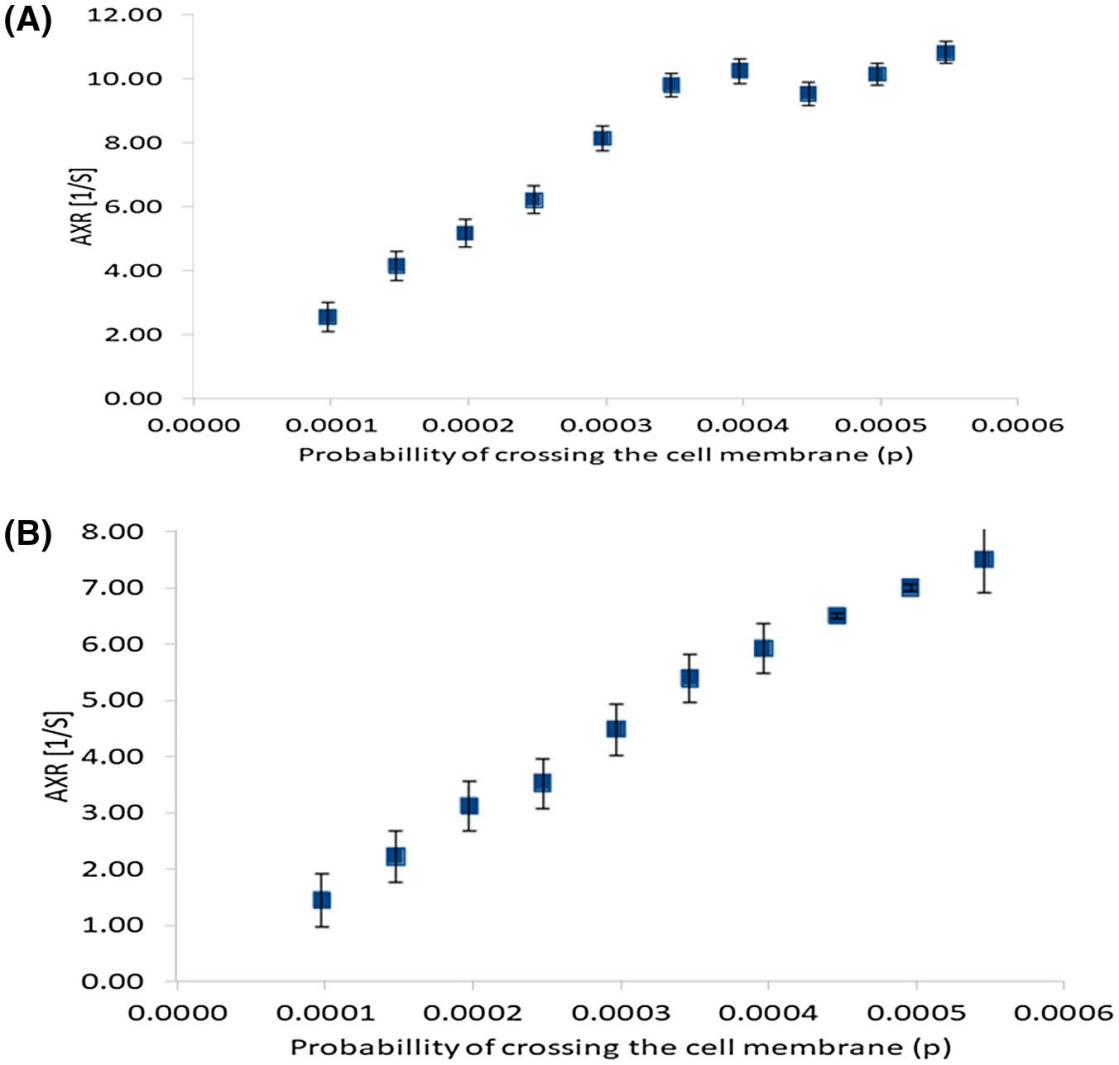
Results of FEXSY simulations, displayed as plots of AXR against permeability, for A, the nerve tissue substrate and B, the yeast substrate. Permeability values correspond to the range in which diffusion exchange peaks could be observed in DEXSY diffusion‐diffusion plots

Figure 4A shows AXR plotted against permeability in the range *P* = .0001 to .00055 for the nerve tissue substrate, although it is not obvious the relationship is monotonic, with a Spearman’s rank correlation coefficient of 1 (*P* < .05).However, it seems to break down at between *P* = .0004 and .0005. Conversely, for the yeast substrate in the range *P* = .0001 to .00055, this relationship is clearly monotonic with a Spearman’s rank correlation coefficient of 1 (*P* < .05) (Figure 4B).

### 3.2 In vitro experiments in yeast

**Figure 5:**
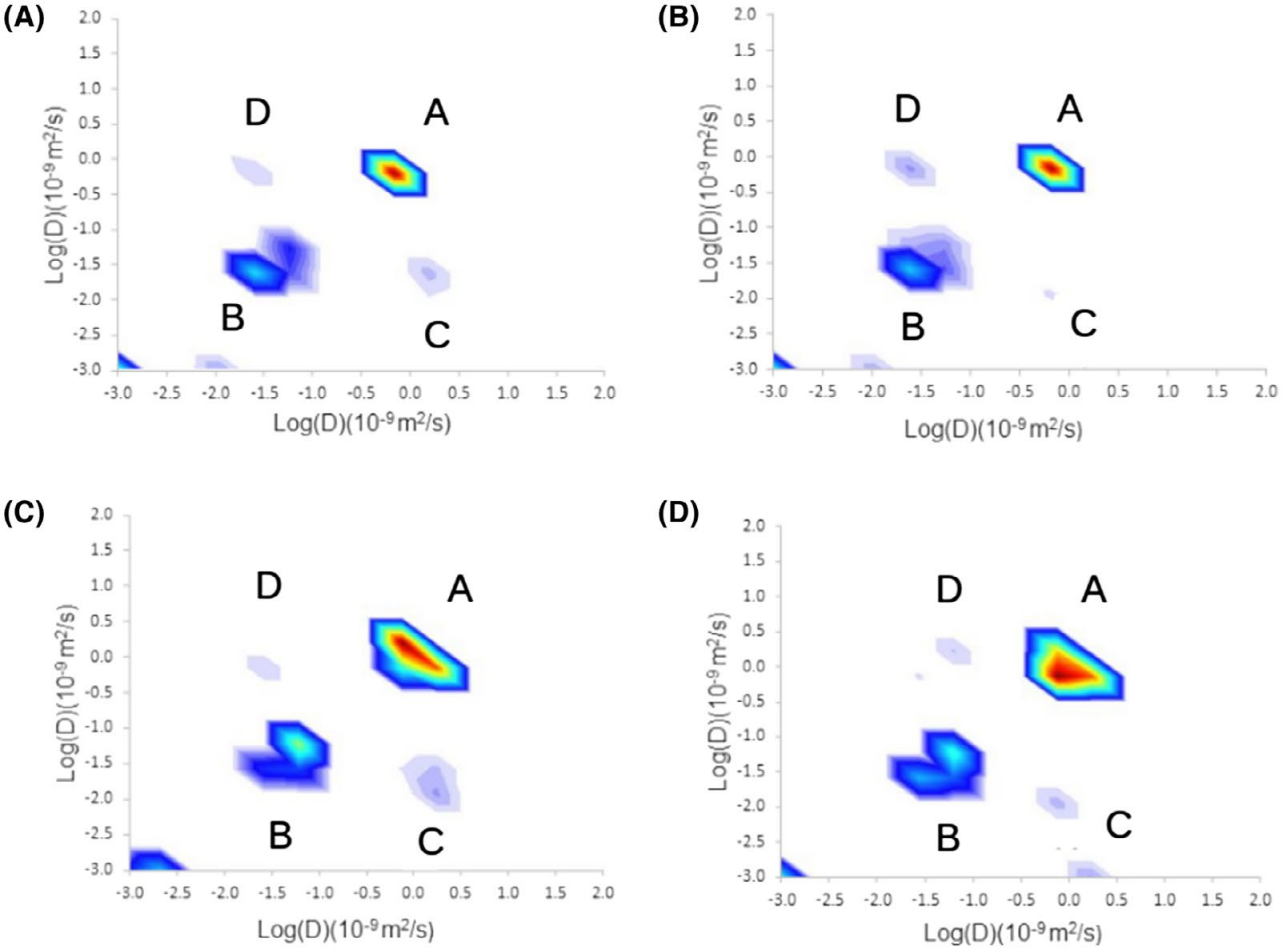
DEXSY diffusion‐diffusion plots calculated from in‐vitro measurements in yeast, at baseline (*t* = 0) in sample 1 (A), and at *t* = 102 min (B). The same plots are shown from sample 2 at baseline (C) and at *t* = 102 min (D). Sample 1 contained a lower concentration of yeast (18 g in 10 mL PBS) than sample 2 (22 g in 10 mL PBS). Peaks A‐D are labeled according to the convention defined above

Figure 5A,B show the results of in vitro DEXSY measurements from sample 1 (the lower yeast concentration). Extracellular (A) and intracellular (B) diffusion peaks, alongside diffusion exchange peaks (C and D) can be identified at baseline (acquisition 1) and at 102 minutes later (acquisition 2). Intracellular diffusivity measurements were larger than in simulations (0.32 ± 0.006) × 10 ${}^{-9}\;m^{2}$/s vs (0.06 ± 0.006) × 10 ${}^{-9}\;m^{2}$/s, potentially due to differences in yeast cell size.

We measured DEI= 0.045 for the first acquisition and also DEI = 0.045 for the second acquisition (quoted to 2 significant figures). Figures 5C,D show DEXSY scans from sample 2, which contained a higher concentration of yeast than sample 1. Again, extracellular, intracellular, and diffusion exchange peaks were present in both acquisitions and, for the first scan DEI = 0.039, and again DEI = 0.039 for the second scan (to 2 significant figures). Values of DEI for the first and second scans for both samples are consistent suggesting good repeatability.

### 3.3 In vivo experiments in mouse tumor xenograft models

**Figure 6:**
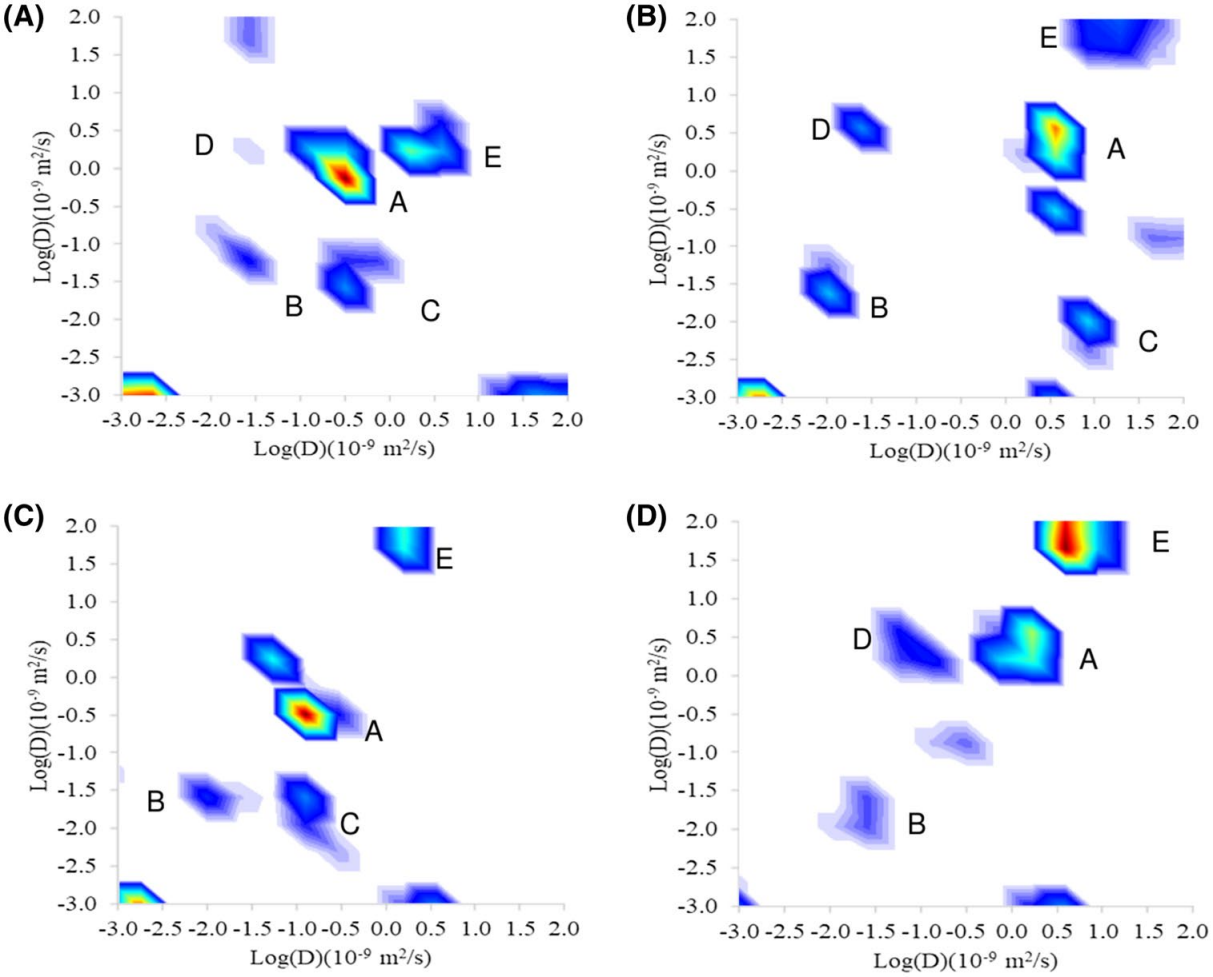
Diffusion‐diffusion exchange plots from a DEXSY measurements performed in a murine tumor xenograft model (subcutaneous) at an initial time point (A) and ten days later (B). C, and D, show DEXSY diffusion‐diffusion plots at a single time point in two further mice, with tumors derived from the same cell line. Potential diffusion exchange peaks are labeled C and D while, potential extracellular diffusion, intracellular diffusion, and perfusion peaks, are labeled A, B, and E, respectively

Figure 6A,B show DEXSY diffusion‐diffusion exchange plots from the same subcutaneous tumor, scanned at an initial time point and then again 10 days later. Between the two time points, the tumor volume increase by a factor of 1.6, which could in part explain differences between exchange plots. Figure 6C,D show DEXSY diffusion‐diffusion exchange plots from tumors (derived from the same cell line) in two different mice. In each of the diffusion‐diffusion plots in Figure 6, potential diffusion exchange peaks are labeled C and D, while potential extracellular, intracellular, and perfusion peaks are labeled A, B, and E, respectively. The addition of a peak corresponding to vascular perfusion appeared at very high diffusivity ( $>1.0\;\times \;10^{-8}\;m^{2}$/s). Previous work using VERDICT MRI,^29^ provided guidance on the diffusivity to expect for intracellular, extracellular, and vascular pseudo‐perfusion in the same type of subcutaneous xenograft model. As such, perfusion peaks representing the pseudo‐diffusion in the microvasculature appeared where expected. The DEIs for the four scans are 0.26, 0.91, 0.30, 0.23 (to 2 significant figures). There is great variability in DEI covering a similar range to that found in the yeast substrate simulations. However, a great deal of variability could be observed between scans, which could reflect the variation in the size and shape of the tumors, alongside physiological variation and measurement uncertainty.

## 4 DISCUSSION AND CONCLUSION

In this study, we have presented numerical simulations, in vitro and in vivo data that demonstrate the feasibility of measuring diffusion exchange across the cell membrane with DEXSY.

Peaks corresponding to diffusion exchange were observed in numerical simulations, for a wide range of physiologically relevant cell membrane permeabilities (1.0 μm/s‐1.4 μm/s). Moreover, there is a clear monotonic relationship between cell membrane permeability and the DEI parameter, both in nerve tissue and yeast substrates. Conversely, the FEXSY AXR parameter displayed a less clear relationship with permeability. However, the calculation of the inverse Laplace transform is affected by the diffusion exchange rate and restriction effects during each PGSE block, which result in non‐Gaussian diffusion,^12^, ^30^ and this is likely to be responsible for the deviation of the results from the expected linear relationship between DEI and permeability.

Results of our in vitro experiments in yeast suspensions revealed intracellular and extracellular diffusion peaks, alongside exchange peaks. These peaks are observed with two different sets of DEXSY scan parameters, which are sensitive to two different diffusion lengths. The DEI measured in vitro, with the two scans was 0.039 and 0.045, which is within the range of DEI measurements found in silico. This reflects our in silico findings. There is a noticeable difference in the size of the peaks observed in the two samples; however, this can easily be explained by the 20% difference in the concentration of the yeast suspension used in each sample. However, further work could be done to determine if the relationship between DEI and permeability found in silico, is also found in‐vitro. This could be achieved by conducting an experiment in which the permeability of the yeast is altered with a detergent (or similar).^13^ in silico and in vitro experiments provided a basis for interpreting the in vivo measurements in mouse tumor xenograft models. These are the first DEXSY data to be acquired in vivo, and provided some challenges in their interpretation. There was evidence of intracellular and extracellular compartments, in addition to diffusion exchange and perfusion peaks, although with much greater variability than in in‐vitro measurements. It is difficult to determine how the differences in tumor microstructure between the different scans relate to the variations in the DEXSY signal acquired. This is because the tumors are likely to vary in terms of cell size, density, necrotic fraction, and vascular perfusion. However, it is also clear that the variations in the signal acquired could indicate that the technique is highly sensitive to changes in tissue microstructure.

Further in vivo validation is needed to determine whether DEI can be used as a quantitative indicator of cell membrane permeability. A significant advantage of DEXSY is that it provides a model‐free approach to measuring diffusion exchange, and, as suggested by our numerical simulations, the DEI is perhaps more directly related to membrane permeability than AXR from FEXSY. A limitation of this study is the low spatial resolution and further work using rapid imaging techniques,^31^ to investigate the influence of heterogeneous tumor pathology (such as necrosis, edema, cysts), and phenomena such as changes in cell size and/or viscosity on DEI measurements should be undertaken. The hardware requirements for DEXSY and FEXI are very similar so it should be possible to implement DEXSY in clinical scanners. However, DEXSY scans would inherently require longer acquisition times than FEXSY, which could limit clinical translation.

Our DEXSY acquisitions currently take 102 minutes, using a comprehensive acquisition consisting of a 16 × 16 acquisition matrix. However, work has been published which advocates a new method for reducing the acquisition time. The MADCO framework restrains the acquisition parameters based on a 1D diffusion spectra in order to reduce the number of data points acquired resulting in potentially more robust data processing.^31^, ^32^ This technique could remove spurious peaks and reduced the acquisition time required to image the whole human brain with DEXSY to 22 minutes,^31^ which makes DEXSY a viable technique for in‐vivo imaging in humans.
